# A Formalisation of Aristotle’s Assertoric Syllogistic in Isabelle/HOL

**DOI:** 10.1007/s11245-025-10184-6

**Published:** 2025-04-18

**Authors:** Angeliki Koutsoukou-Argyraki, Karol Wapniarski

**Affiliations:** 1 Department of Computer Science, Royal Holloway, University of London, Egham, UK; 2 Department of Computer Science and Technology, University of Cambridge, Cambridge, UK; 3 Faculty of Psychology and Cognitive Science, Adam Mickiewicz University, Poznań, Poland

**Keywords:** Aristotle's syllogistic, Existential import, Isabelle/HOL, Proof assistants, Interactive theorem proving, Formal proofs, Higher-Order Logic

## Abstract

We present a formalisation of Aristotle’s Assertoric Syllogistic in the proof assistant (interactive theorem prover) Isabelle/HOL and discuss the insights gained by the formalisation, as we demonstrate that the formal proofs and especially Isabelle’s automation can enhance the user’s understanding and facilitate metatheoretical explorations.

## Introduction

Proof assistants (interactive theorem provers) are tools used in formal verification, which involves proving the correctness of computer software or hardware, showing properties of programming languages and protocols, and also verifying mathematical proofs via formalisation. To this end, mathematical formulas are expressed in a formal language and proved in a logical calculus with the help of various automated reasoning tools.

Isabelle^1^ is a generic proof assistant originally developed by Lawrence C. Paulson, Tobias Nipkow and Makarius Wenzel, starting with early work by Paulson from the late 1980’s (Paulson (1989)). Isabelle/HOL (Nipkow et al. (2002)), which is based on Higher-Order Logic, is the most widely used instance of Isabelle, and one of the most popular proof assistants for the formalisation of mathematics nowadays. It is based on simple types, allows for proofs in classical logic, and includes the Axiom of Choice. Proof scripts in Isabelle are interactive sessions between user and theorem prover, thus enabling interactive development of verifiable proofs with the help of integrated automated reasoning tools. Isabelle features structured, and thus easily readable, proofs. Thanks to its underlying simple type theory, Isabelle/HOL features efficient automation, which is much more powerful than the automation featured in any other proof assistant: Its main automation tool, Sledgehammer (Blanchette et al. (2024)), calls a number of external automated theorem provers that can provide a proof, each of the provers giving either the same or possibly different proofs, for (simple enough) proof goals. Isabelle/HOL is moreover supported by counterexample-finding tools such as Nitpick and Quickcheck.

The Archive of Formal Proofs^2^ has been hosting, since 2004, a vast and rapidly growing online collection of formalised material building on the Isabelle libraries. As of 15 July 2024, there are 837 entries by 502 authors. While the main areas of focus are computer science, mathematics, and logic, there is also some formalised material in the area of philosophical logic too. In particular, there are several contributions in philosophical logic by Christoph Benzmüller, David Fuenmayor and others.^3^

In this work, we present a formalisation of Aristotle’s Assertoric Syllogistic in the proof assistant Isabelle/HOL by the first author (the code can be found on the Archive of Formal Proofs (Koutsoukou-Argyraki (2019))), based on the article from the Stanford Encyclopedia of Philosophy by Robin Smith (Smith (2019)). We also present some recent additions and exploration by the second author who learned about this work at the recent 14th Panhellenic Logic Symposium, where it was presented as part of the first author’s invited tutorial (Koutsoukou-Argyraki (2024)), and took an interest in expanding it, as well as a discussion on the insights gained by the formalisation. We hope that this work would illustrate how proof assistants like Isabelle/HOL can *actually assist* users in their understanding of proofs and, in the case at hand, of philosophical arguments too. At the same time, Aristotle’s Logic in the 21st century remains a source of inspiration especially for developments in cognitive modelling and computational argumentation in Artificial Intelligence (Saldanha and Kakas (2019)), so this formalisation of Aristotle’s Assertoric Syllogistic can be seen as a pilot study.

The plan of this paper is as follows: In Section 2, we present the formalisation in Isabelle/HOL of Aristotle’s universal, particular and indefinite predications (affirmations and denials) expressed using a set theoretic formulation. Based on these definitions, we then show the proofs of the three main conversion rules as well as of all the deductions (“Moods”) in the three Figures. In Section 3, we present some supplementary formalisations, namely the two subalternations and the formalisation of the deductions in the Fourth Figure (implicit in Aristotle but studied extensively by later scholars) following material from Parsons (2011) and the first section of Lagerlund (2022). In Section 4, we show how we can reproduce Aristotle’s metatheorem that all deductions in all Figures can eventually be reduced to either one of two specific deductions, in particular to either Barbara or Celarent, we discuss the issue of existential import among several other noteworthy observations that emerged from the formalisation, and we comment on the insights gained, before concluding in Section 5.

## The Formalisation

### Introductory Concepts and the Square of Opposition

Aristotle’s Logic (Parry and Hacker (1991)) is considered the first formal study of logic. A central notion in Aristotle’s Logic is the concept of *syllogism* (deduction):

“A deduction is speech (*logos*) in which, certain things having been supposed, something different from those supposed results of necessity because of their being so” (Prior Analytics I.2, 24b18–20),

so *logos* can be translated as *reasoning*. As explained by Smith (2019), syllogisms, according to Aristotle, are “structures of sentences each of which can meaningfully be called true or false”. Every such sentence (*assertion*) must contain a subject and a predicate and must either affirm or deny the predicate of the subject. Thus, every assertion is either the *affirmation* or the *denial* of a single predicate of a single subject. The subject may be either individual or universal, however the predicate may be only universal, and, as Robin Smith explains (Smith (2019)), Aristotle treats individual predications and general (i.e. universal) predications as similar in logical form, e.g. we may say “Socrates is an animal” and “Humans are animals” (but we may not say “Socrates is George”, i.e. “animal”/ “animals” in the aforementioned predications mean “the set of animals” which, in modern terminology, should not be a one-element set). In the case where the subject is a universal, predication can be either universal or particular. We have the following combinations, presented in Table 1 with the corresponding abbreviations in Table 2 (here we have slightly modified the notation followed by Smith (2019) so that it will match the notation used in our Isabelle code (Koutsoukou-Argyraki (2019))).

**Table 1:** Combinations including the Square of Opposition

	Affirmations	Denials
Universal	P affirmed of all of S (Every S is P)	P denied of all of S (No S is P)
Particular	P affirmed of some of S (Some S is P)	P denied of some of S (Some S is not P)
Indefinite	P affirmed of S (S is P)	P denied of S (S is not P)

The universal affirmation, universal denial, particular affirmation and particular denial constitute the so-called *Square of Opposition*. The formalisation in Isabelle/HOL is given in code screenshot 1.

**Fig. 1:**
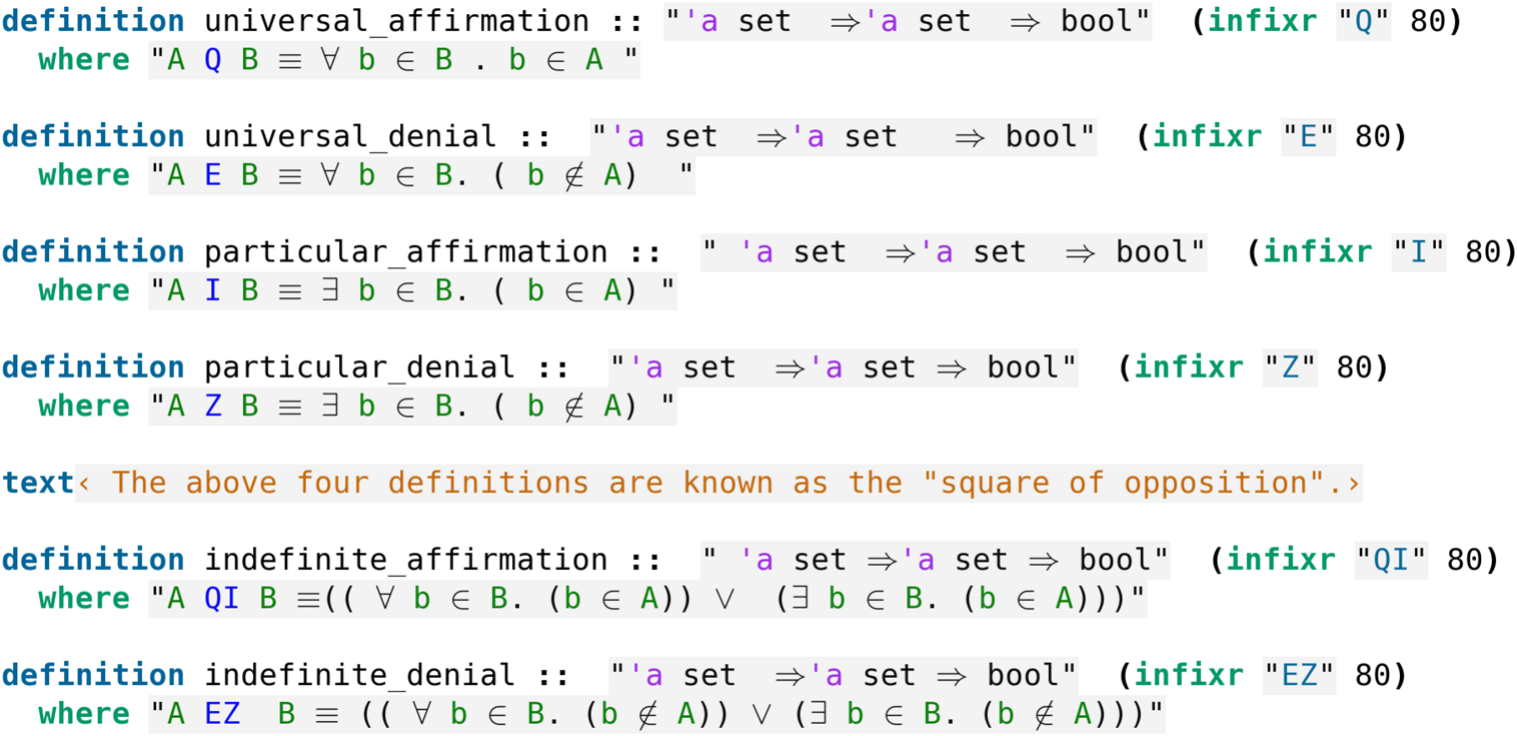
Formalisation of the sentences

**Table 2:** Sentences with abbreviations

**Abbreviations**	Sentences	Classification
AQB	A belongs to all B (Every B is A)	Universal affirmation
AEB	A belongs to no B (No B is A)	Universal denial
AIB	A belongs to some B (Some B is A)	Particular affirmation
AZB	A does not belong to all B (Some B is not A)	Particular denial
A(QI)B	B is A	Indefinite affirmation
A(EZ)B	B is not A	Indefinite denial

The indefinite sentences are unspecified, hence implemented with an $\vee$ (or). We remind that the predicate can only be universal so Aristotle would never consider A as a one-element set. Aristotle noted the conversions AEB$\rightarrow$BEA, AIB$\rightarrow$BIA, AQB$\rightarrow$BIA which are proved in Isabelle/HOL as shown in code screenshot 2.

**Fig. 2:**
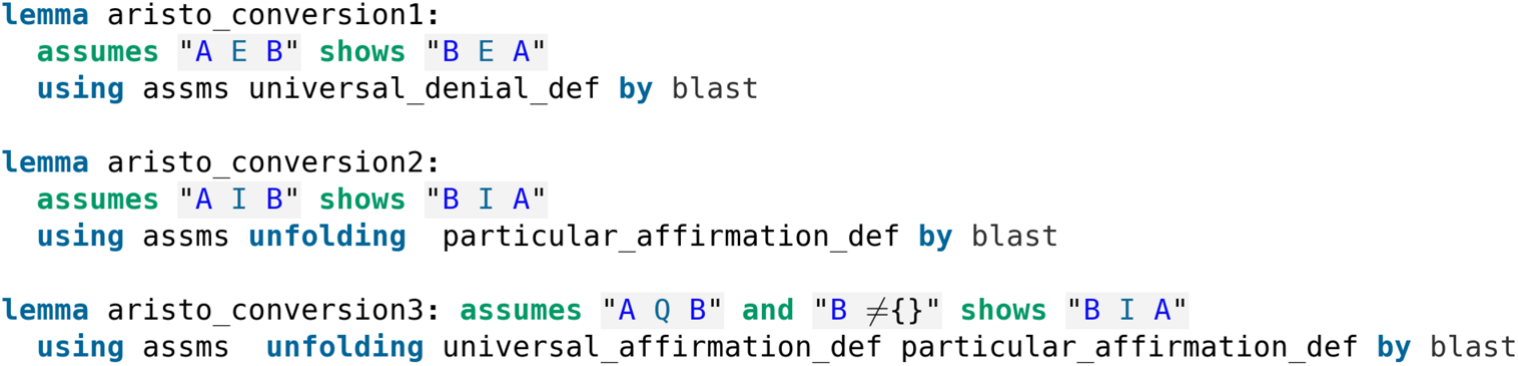
Formalisation of the conversions

The proofs were found automatically by calling Sledgehammer, Isabelle’s automation tool, and it can be seen that they make use of the respective assumptions (assms) and relevant definitions as previously introduced, and they apply Isabelle’s blast automatic proof method.

### The Deductions (Moods) of the Figures

As explained by Smith (2019), in *Prior Analytics* Aristotle systematically investigates all possible combinations of two premises in each of the three Figures, and shows that the premise combinations given in each table for the respective Figure yield certain deductions, while the other premise combinations fail to yield any deduction (Table 3). For Aristotle’s arguments we use the traditional terminology (mnemonic names) used by scholars since the middle ages as followed by Smith (2019).

Aristotle distinguishes between *perfect* and *imperfect* deductions. The deductions which are called perfect do not use another external deduction for their proof, while the imperfect deductions do (Smith (2019)). Aristotle moreover distinguishes between two types of proof, the *direct* and the *indirect* (*by impossibility*) proof. The first one is used for almost every Mood and consists of a series of steps leading from the set of premises to a conclusion, where each of the steps consist of either applying one of the conversions or of applying (one of the) other deductions (Smith (2019)). Proof by impossibility is slightly more intricate and is used less frequently. We explain proof by impossibility in detail in Sect. 4.1.

**Table 3:** Figure pattern

	First Figure	Second Figure	Third Figure
	Predicate, subject	Predicate, subject	Predicate, subject
Premise	A, B	A, B	A, C
Premise	B, C	A, C	B, C
Conclusion	A, C	B, C	A, B

**Fig. 3:**
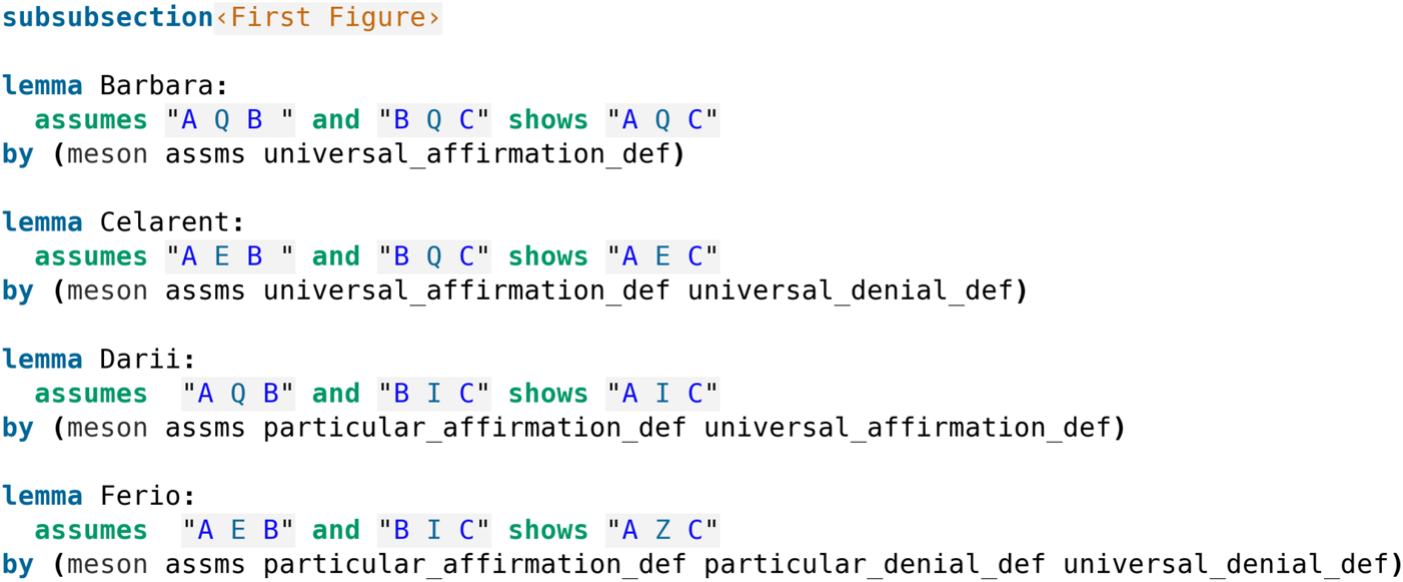
Formalisation of the First Figure

The proofs in our formalisation (Koutsoukou-Argyraki (2019)) have been automatically found by calling Sledgehammer. The deductions of the First Figure (Table 4) are described as perfect, and, indeed, we observe that the formal proofs found automatically use only the definitions of the sentences, without making any use of other deductions. In our code screenshot 3 we see that Isabelle’s proof method meson is employed (but other Isabelle proof methods could have been alternatively used to reason using the definitions of the sentences).

**Table 4:** First Figure

AQB, BQC $\vdash$ AQC	Barbara	Perfect
AEB, BQC $\vdash$ AEC	Celarent	Perfect
AQB, BIC $\vdash$ AIC	Darii	Perfect; also by impossibility, from Camestres
AEB, BIC $\vdash$ AZC	Ferio	Perfect; also by impossibility, from Cesare

Now let us look at an elementary example. In our code screenshot 4, we see an elementary example of a deduction with general (i.e., universal) predication, more specifically we have an instance of the deduction Barbara: “Every human is mortal, and every Greek is human, therefore every Greek is mortal”. Isabelle’s automation immediately sees how to prove this, as the proof simply involves Barbara (here previously formalised as a lemma in Isabelle, see code screenshot 3), the assumptions (assms), and the use of the basic automatic proof method auto. Furthermore, in our example of a deduction with individual predication, we consider “Socrates” to be a set element, rather than a set. To show the deduction: “Every human is mortal, and Socrates is human, therefore Socrates is mortal”, Isabelle’s automation here simply uses the assumptions, the definition of universal affirmation, and applies its basic automatic proof method simp which is enough to give a proof.

**Fig. 4:**
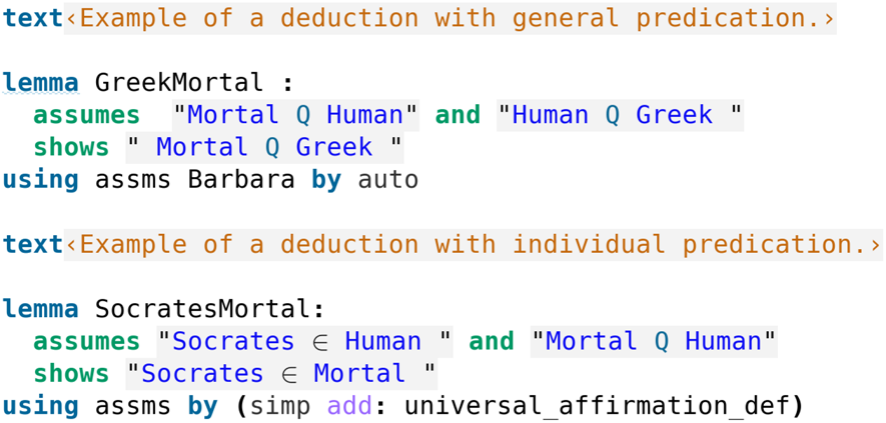
Example

The Second Figure is, at first glance, slightly more involved, as here none of the deductions are described as “perfect”. Interestingly, we observe that most of the proofs found by Isabelle’s automation (see code screenshot 5) reflect exactly the proofs described by Robin Smith in Table 5, with the exception of Baroco. In particular, Cesare is shown by applying the first conversion rule (AEB $\rightarrow$ BEA) and Celarent, which matches the indicated proof. For Camestres, the indicated proof involves applying the first conversion rule (twice: AEC $\rightarrow$ CEA and CEB $\rightarrow$ BEC) and using Celarent. The proof by Isabelle, in addition to the conversion, uses Cesare instead of Celarent, but we have just seen that Cesare reduces to Celarent. For Festino, the indicated proof involves applying the first conversion rule and using Ferio, which matches the proof discovered by Isabelle’s automation. Baroco is more interesting: even though an indirect proof (proof by impossibility; we explain the format of proof by impossibility in Section 4.1) and an application of Barbara is indicated, Isabelle finds a proof that is simpler and not making use of any other deductions, involving simply applying the definitions of the sentences and using the automatic proof method meson, thus Baroco appears as a perfect deduction too, just like the deductions of the First Figure (compare with code screenshot 3). For the rest of the formal proofs, here the basic Isabelle automatic proof method blast is used (but other too could also work).

**Table 5:** Second Figure

AEB, AQC $\vdash$ BEC	Cesare	(AEB, AQC) $\rightarrow$ (BEA, AQC)$\vdash _{Celarent}$ BEC
AQB, AEC $\vdash$ BEC	Camestres	(AQB, AEC) $\rightarrow$ (AQB, CEA) $\vdash _{Celarent}$ CEB $\rightarrow$ BEC
AEB, AIC $\vdash$ BZC	Festino	(AEB, AIC) $\rightarrow$ (BEA, AIC) $\vdash _{Ferio}$ BZC
AQB, AZC $\vdash$ BZC	Baroco	(AQB, AZC (+ BQC))$\vdash _{Barbara}$(AQC, AZC) $\vdash _{Impossibility}$ BZC

**Fig. 5:**
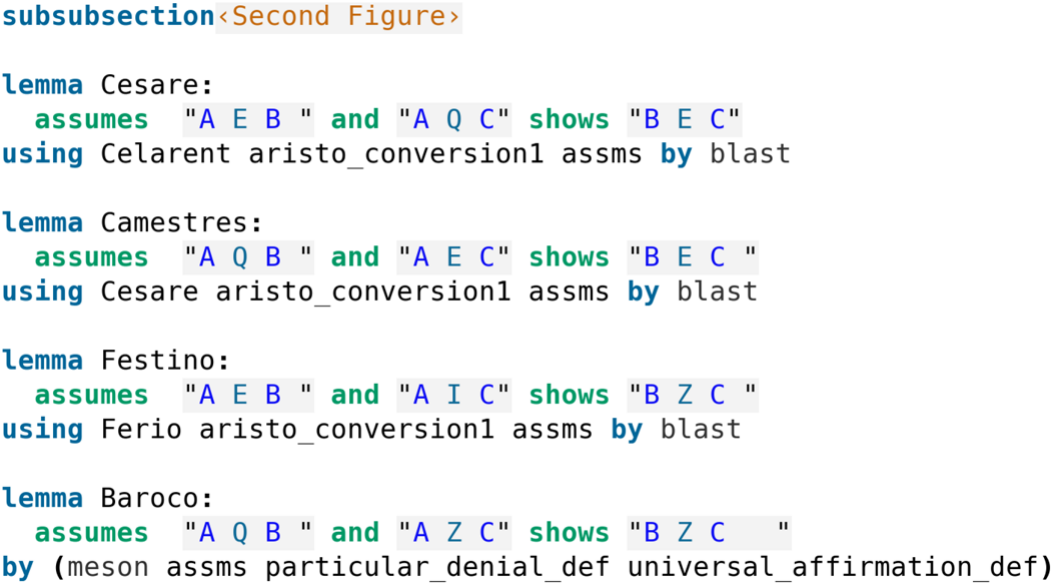
Formalisation of the Second Figure

We can make some similar observations about the deductions of the Third Figure (compare Table 6 and code screenshot 6): The indicated proof for Darapti uses the third conversion rule as well as Darii, while the proof by Isabelle in addition to Darii does not make the application of the conversion rule explicit, rather uses the definitions that had been used for the proof of the conversion rule (see code screenshot 2). The indicated proof for Felapton in addition to a conversion rule uses Ferio, while the Isabelle proof uses Festino; we have however seen that Festino reduces to Ferio (see code screenshot 5). The indicated proof for Disamis uses Darii alongside one of the conversion rules, which is exactly reproduced in the given Isabelle proof. The indicated proof for Datisi is similar, while the Isabelle proof, instead of using Darii, it uses Disamis, but we have just seen that Disamis indeed reduces to Darii. Interestingly, while the indicated proof for Bocardo (via impossibility, and an application of Barbara), is more involved, Isabelle finds a proof that is simpler and does not use any other deductions, involving simply applying the definitions of the sentences and using the automatic proof method meson (recall this was exactly the case for Baroco in the Second Figure too), thus Bocardo too appears as a perfect deduction, just like Baroco and the deductions of the First Figure (compare code screenshot 6 with code screenshots 3 and 5). Finally, the indicated proof for Ferison uses Ferio and the second conversion rule, which is exactly the proof shown here by Isabelle too. The careful reader may have observed (code screenshot 6) that Darapti and Felapton are the only deductions that require a nonempty set assumption, alongside the third conversion rule (code screenshot 2). We will return to this important observation in the discussion (Section 4). It is moreover important to note that, as we will explain in Sect. 4, several alternative direct proofs are possible for almost all deductions (Wapniarski and Urbański (2024a)) and we have actually verified that all of them can be found by Isabelle. Furthermore, we have already commented on certain deductions being reducible to others as it is evident by looking at the formal proofs discovered by Isabelle, and in the discussion section (Sect. 4) we will fully show how the conclusion that all deductions can be reduced to either Barbara or Celarent, a metatheoretical conclusion noted by Aristotle, can emerge from Isabelle’s formal proofs.

**Table 6:** Third Figure

AQC, BQC $\vdash$ AIB	Darapti	(AQC, BQC) $\rightarrow$ (AQC, CIB)$\vdash _{Darii}$ AIB
AEC, BQC $\vdash$ AZB	Felapton	(AEC, BQC)$\rightarrow$ (AEC, CIB) $\vdash _{Ferio}$ AZB
AIC, BQC $\vdash$ AIB	Disamis	(AIC, BQC) $\rightarrow$ (CIA, BQC)$\vdash _{Darii}$ BIA $\rightarrow$ AIB
AQC, BIC $\vdash$ AIB	Datisi	(AQC, BIC) $\rightarrow$ (AQC, CIB)$\vdash _{Darii}$ AIB
AZC, BQC $\vdash$ AZB	Bocardo	(AZC, +AQB, BQC)$\vdash _{Barbara}$ (AQC, AZC) $\vdash _{Impossibility}$ AZB
AEC, BIC $\vdash$ AZB	Ferison	(AEC, BIC) $\rightarrow$ (AEC, CIB) $\vdash _{Ferio}$ AZB

**Fig. 6:**
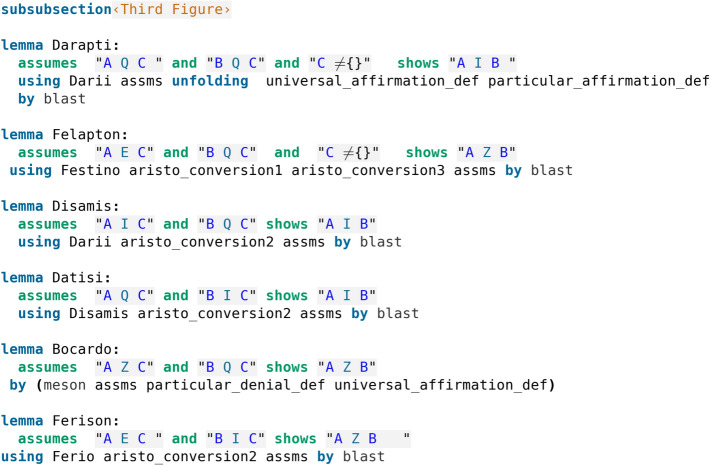
Formalisation of the Third Figure

## Extension of the Formalisation

In this section, we give an extension of the above presented development (Koutsoukou-Argyraki (2019)), including the two subalternation principles, subalternated Moods, and the Fourth Figure.

### Subalternations

Subalternations were not explicitly noted by Aristotle himself, but are in his writings by implication (Parsons (2011)) and form an essential part of the Square of Opposition. They consist of deriving a particular conclusion from a general (i.e. universal) one (AQB$\rightarrow$AIB, AEB$\rightarrow$AZB) and were easily proved in Isabelle/HOL by calling Sledgehammer (code screenshot 7).

**Fig. 7:**
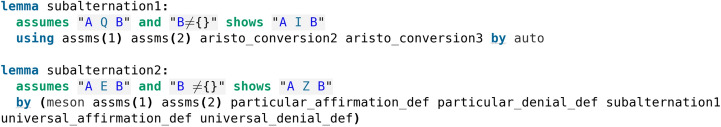
Subalternations

After recognizing the subalternations, several additional deductions (traditionally referred to as the subalternated Moods) can be added to the Figures, stemming from subalternating a general conclusion of a syllogism and getting a particular one. In this way, the First Figure gets additional Barbari from Barbara and Celaront from Celarent. The Second Figure has Cesaro and Cemestros added (from Cesare and Camestres), and the Third Figure stays the same as every Mood it contains already has a particular conclusion. All the additional Moods were easily proved by Isabelle’s automation. In code screenshot 8, we can see that Barbari and Celaront are proved by applying Barbara and Celarent respectively together with the subalternation principles as one would expect. Cesaro and Camestros in turn are not proved by Cesare and Camestres, but instead a simpler proof by Celarent was found (recall that he have indeed already seen from the formal proofs in code screenshot 5 that Cesare and Camestres reduce to Celarent).

**Fig. 8:**
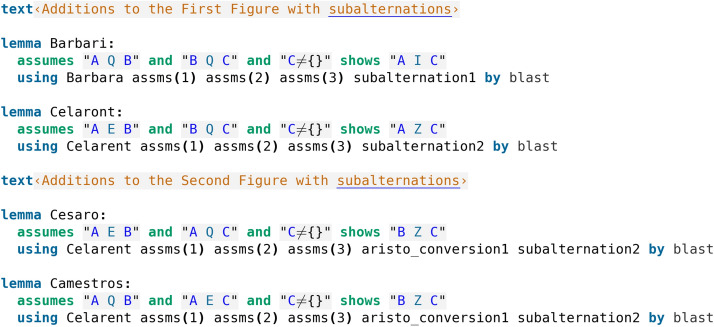
Additional deductions in the First and Second Figure stemming from the subalternations

### Fourth Figure

The Fourth Figure is not explicitly recognized by Aristotle as a separate Figure, although the Moods it contains are present in his writings as the indirect Moods of the First Figure. The form of the Fourth Figure follows the reversed pattern for the premises of the First Figure, so the predicate-subject arrangement becomes B, A and C, B for the premises and stays A, C for the conclusion (compare with Table 3 in Section 2.2.). Historically, the Fourth Figure Moods were discussed in detail by the first commentators of Aristotle (Lagerlund (2022)), as well as by medieval European (Sabra (1965)) and Arabic (Rescher (1965)) scholars. Here, we follow the mnemonic names from the most widespread version presented for example in Aldrich (1862) (see also Lagerlund (2022), but note that it uses the names Camestrop instead of Camestros and Camenop instead of Camenos).

With the subalternation principles and the Fourth Figure added, we obtain a complete formalisation of all 24 classically recognized valid syllogistic Moods. The formalisation of the Fourth Figure is presented in code screenshot 9. Here, three proofs found by Isabelle reflect the prescribed Aristotelian reductions (Camenes is proved by Celarent, Dimaris by Darii, and Fresison by Ferio). The proof found for Bramantip uses conversion and Barbari instead of Barbara, but we have seen in the previous subsection that Barbari reduces to Barbara (see code screenshot 8). The proof for Fesapo uses Festino which can be further reduced to Ferio (see code screenshot 5), and the proof for Camenos (note that Camenos is the subalternated form of Camenes) uses subalternation and Cesare which is reducible to Celarent (see code screenshot 5). We can therefore see that again, Isabelle either directly applies the perfect First Figure Moods or finds alternative proofs via Moods which are ultimately reducible to the First Figure ones. For the first three Figures we have already noted that we have actually moreover verified that all possible proofs for each deduction are discoverable by Isabelle, and this is the case also for the deductions in the Fourth Figure. We elaborate more on this in the discussion section (Sect. 4). A careful reader may also again observe that some of the Moods require the assumption that certain sets are nonempty. We will explain this observation in detail in Sect. 4.

**Fig. 9:**
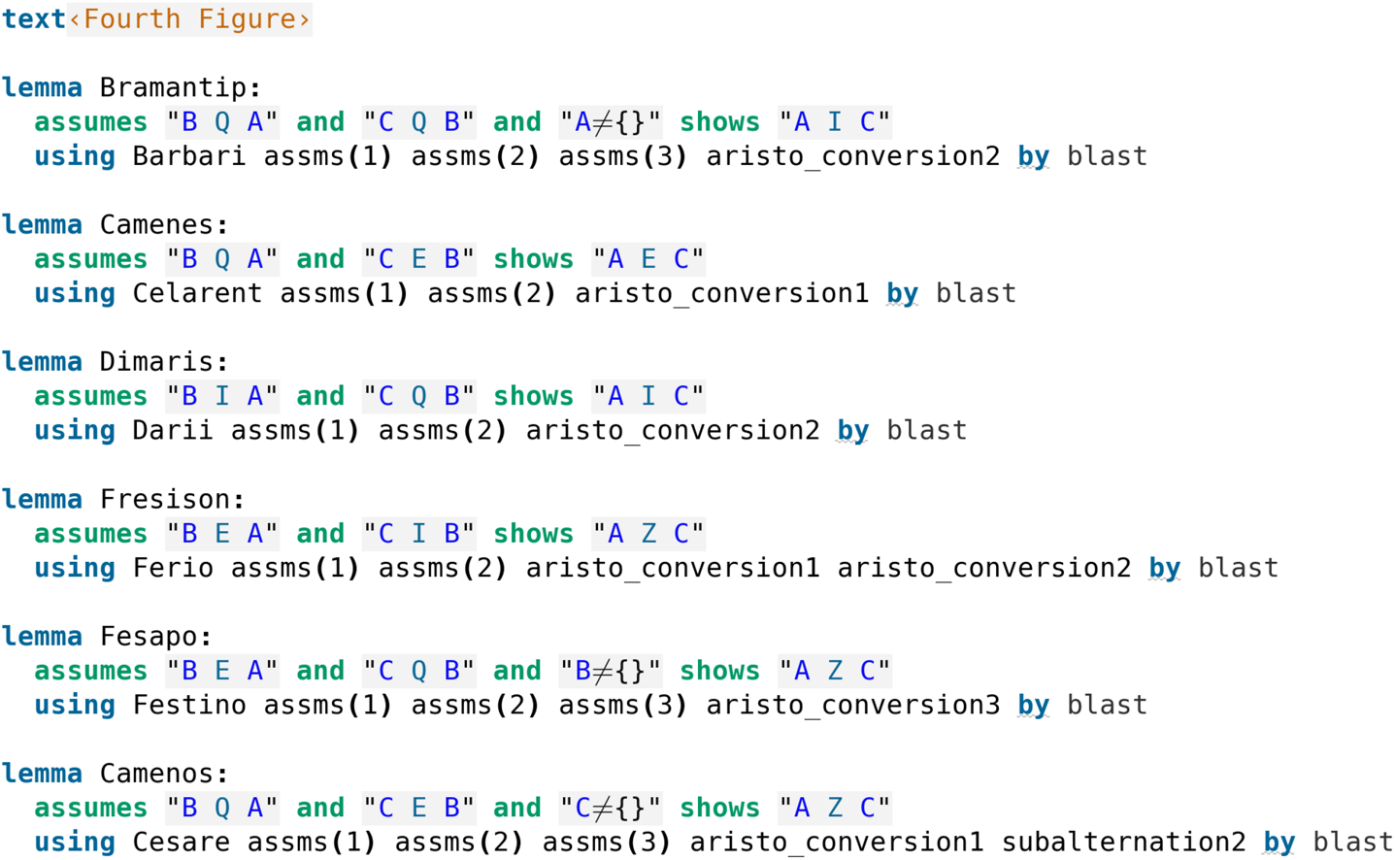
Formalisation of the Fourth Figure

## Discussion

In this section, we discuss some interesting observations that emerged from the formalisation.

### Aristotle’s Metatheorem on Reducing Deductions

As we have already commented, certain deductions are reducible to others as it is evident by looking at the formal proofs discovered by Isabelle (see code screenshots 3, 5 and 6). Aristotle had actually observed that all deductions in the Figures can be reduced to either Barbara or Celarent (Smith (2019)). We moreover show some of the reductions of the deductions that have not been directly evident by the formal proofs so far. To this end, we develop formal proofs in Isabelle/HOL following exactly Aristotle’s “by impossibility” method as explained by Smith (2019). The idea is as follows: to show that e.g., Darii is reduced to Camestres, we formulate and prove Darii using a supplementary assumption of the negation of the conclusion and observe that the proof uses Camestres. Following the same method, we reduce Ferio to Cesare, Baroco to Barbara and Bocardo to Barbara. These formal proofs are presented in code screenshots 10 and 11.

**Fig. 10:**
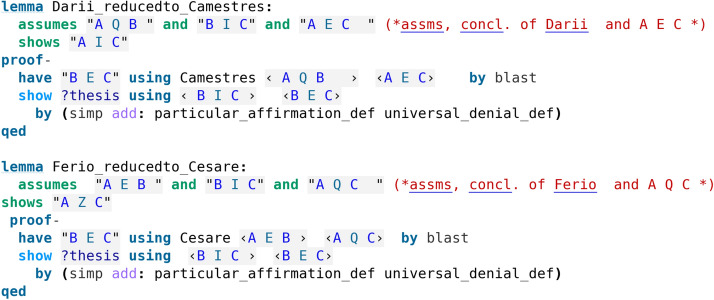
Reduction proofs formalised

**Fig. 11:**
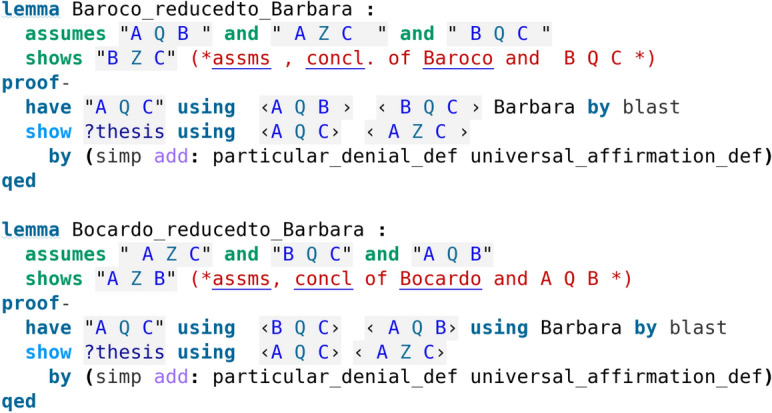
More reduction proofs formalised

To summarise, from all the formal proofs that have been presented, it can be seen that all deductions can be reduced to either Barbara or Celarent via the following chains of reductions (where the arrow here denotes the direction of the reduction):

Baroco $$\hookrightarrow$$ Barbara

Bocardo $$\hookrightarrow$$ Barbara

Felapton $\hookrightarrow$ Festino $\hookrightarrow$ Ferio $\hookrightarrow$ Cesare $\hookrightarrow$ Celarent

Datisi $\hookrightarrow$ Disamis $\hookrightarrow$ Darii $\hookrightarrow$ Camestres $\hookrightarrow$ Cesare

Darapti $$\hookrightarrow$$ Darii

Ferison $$\hookrightarrow$$ Ferio

When it comes to the Fourth Figure, we may further note the following chains of reductions (evident from the proofs in code screenshots 9 and 8) which, combined with the above chains, eventually lead to all deductions being reduced to either Barbara or Celarent:

Bramantip $\hookrightarrow$ Barbari $\hookrightarrow$ Barbara

Camenes $$\hookrightarrow$$ Celarent

Dimaris $$\hookrightarrow$$ Darii

Fresison $$\hookrightarrow$$ Ferio

Fesapo $$\hookrightarrow$$ Festino

Camenos $$\hookrightarrow$$ Cesare

It is interesting that Aristotle’s metatheoretical conclusion can easily emerge from and be reproduced by Isabelle’s formal proofs.

### Existential Import: On the Assumption of Nonempty Sets

Here we address the issue of the so-called “existential import”, that is, whether entities that appear in a syllogism need to exist. The most widespread twentieth-century interpretation stemming from the work of Łukasiewicz (1951) states that Aristotle himself a**ss**umed all the terms in syllogistic to be nonempty. It is however subject of a debate, and there are some voices stating that Aristotle did not explicitly consider this issue at all (Malink (2013)). For sure, the issue has been a matter of debate by scholars of the medieval Arabic world and late-medieval European thinkers and was discussed in detail (Parry and Hacker (1991), Wapniarski (2024)). The most common view advocated by Avicenna (Hodges (2012)) and William of Ockham (Bäck (2000)), among others, states that all true affirmative statements must have an existing subject. From the standpoint of First-Order Predicate Logic, the only Moods which need existential assumptions for their proofs to be valid are those consisting of two general (i.e., universal) statements as premises and a particular statement as a conclusion (Wapniarski and Urbański (2024b)). Apart from Darapti and Felapton in the Third Figure, those include Bramantip and Fesapo from the Fourth Figure and all the subalternated Moods: Barbari, Celaront, Cesaro, Camestros, and Camenos.

The precise term that needs to be nonempty is dependent on the way in which a particular deduction is proved. For First-Figure subalternated Moods that is the subject of the conclusion, as they are proved through subalternations applied on conclusions of the deductions by which they are proved (Barbara by Barbari, Celaront by Celarent). This is the case also for Second-Figure subalternated Moods, as they require subalternating the conclusion of the deduction used in the proof as well. In the Third Figure, the nonempty term is the subject of the premises, as the deductions that require it are proved through applying the third type of conversion (which necessitates nonemptiness of its subject) on either one of these. For example, Felapton is proved by using Festino and applying the third conversion on the second premise. In the Fourth Figure, the term which must be nonempty varies between deductions, which is also determined by their respective proofs. Bramantip which is proved by Barbari requires the same nonempty term as Barbari, but since its premises are switched (note the construction of the Fourth Figure in Sect. 3.2.) that term is not C but A. In the case of Fesapo, the term is the subject of the second premise, as the Mood gets proved by applying the third conversion rule on this premise. An interesting anomaly is Camenos which needs subalternating a general negative conclusion in the proof. Apparently, it is the only Mood in which the nonempty set is not the subject of any of the premises, but a predicate. A careful reader may ask how those observations correspond to the fact that many Moods do have alternative proofs as mentioned at the end of Sect. 2.2. The answer is that all the alternative proofs require the same term to be nonempty. For a more detailed account on this issue consult Urbański et al. (2024).

In the Isabelle/HOL development, for the proofs provided by Isabelle’s automation for the first three Figures we have indeed seen that Darapti and Felapton are the only deductions that require an assumption that the subject is a nonempty set, as it can be seen from code screenshots 3, 5 and 6. Felapton relies on the third conversion rule that requires this assumption, as it can be seen from code screenshot 2. The proof of Darapti displayed here does not explicitly use the third conversion, but it uses the same two definitions (universal affirmation and particular affirmation) that are used for the proof of the third conversion. As we will discuss in Section 4.4, we have found various different alternative direct proofs, which the reader can easily reproduce by repeatedly calling Sledgehammer while making use of the formalisation (Koutsoukou-Argyraki (2019)), and, for the case of Darapti, other alternative proofs do make use of the third conversion or of one of the subalternations (which require nonemptiness). Because, in order to prove both the third conversion rule and Darapti, Isabelle uses the definitions of the universal affirmation and the particular affirmation combined, this appears to be the ultimate source of the nonemptiness of sets assumption in this case, which further agrees with the aforementioned findings present in Wapniarski and Urbański (2024b). For the Fourth Figure, both Bramantip and Fesapo were recognized as requiring the existential assumptions, where Bramantip is proved using the subalternated Barbari and Fesapo using the third conversion rule (see code screenshot 9). Furthermore, all the subalternated Moods also do require the assumption since they are proved using the subalternation which requires it, as can be seen from code screenshot 7.

It can be therefore seen that Isabelle/HOL (which is based on Higher-Order Logic as noted before) has indeed recognized exactly all the cases where existential assumption is needed according to the recent findings in Wapniarski and Urbański (2024b) and Urbański et al. (2024) mentioned above. The rest of the deductions are provable without making use of this assumption, as Isabelle can indeed verify.

In particular, it is interesting to note that in the cases where the assumption that a certain set is not the empty set was necessary, Isabelle’s automation immediately alerts the user that the deduction is false without the nonempty set assumption via its counterexample-finding tool Auto Quickcheck, which gives an explicit counterexample where the set is the empty set. This is illustrated in code screenshot 12 where we have removed the nonempty set assumption for C (compare with the proof of Felapton in code screenshot 6). Here, by attempting to add the assumption that A (instead of C) is nonempty, Auto Quickcheck still gives a counterexample (code screenshot 13), and similarly with attempting to add the assumption that B (instead of C) is nonempty (code screenshot 14). Throughout the entire Isabelle formalisation, wherever a nonempty set assumption has been added, its necessity had been automatically discovered by Auto Quickcheck as in the above described case, with Auto Quickcheck automatically generating counterexamples and the user attempting to add nonempty set assumptions for various sets until the correct nonempty set assumption was found. This shows how Isabelle’s interactive nature and automation can be of substantial help for the user in real time, by providing immediate feedback during the process of formalisation and thus guiding the user.

**Fig. 12:**
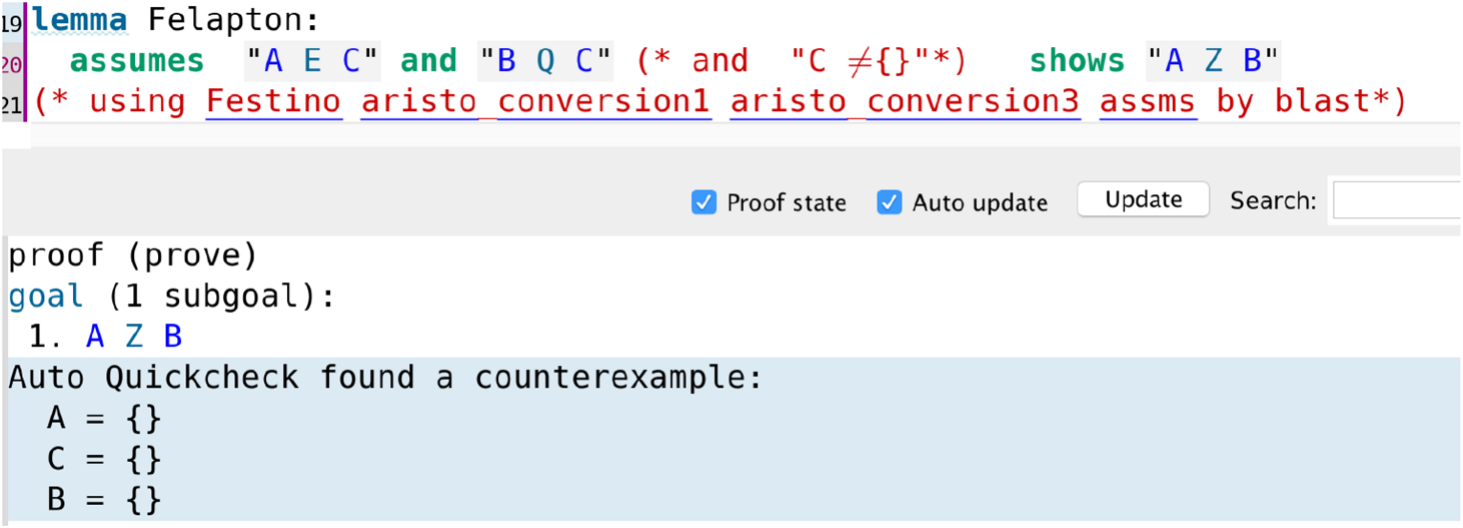
The counterexample-finding power of Isabelle/HOL in action: removing the nonempty set assumption for C yields a counterexample

**Fig. 13:**
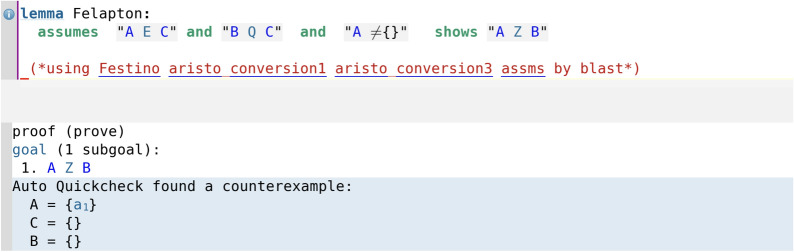
Assuming that A is nonempty (but not C or B) still yields a counterexample

**Fig. 14:**
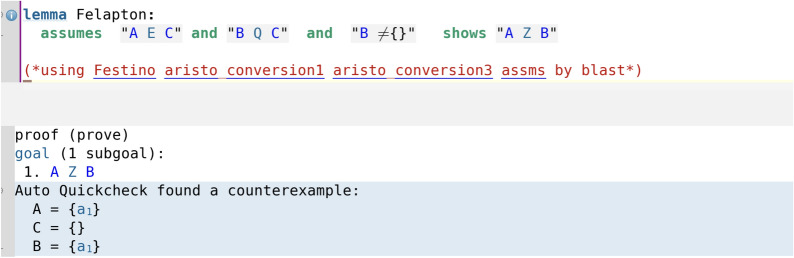
Assuming that B is nonempty (but not C or A) still yields a counterexample

### Baroco and Bocardo Shown by Isabelle/HOL as Perfect Deductions

We have already seen from the above given formal proofs (code screenshots 5 and 6) that for Baroco and Bocardo, even though they belong to the (as we would expect more involved) Second Figure and Third Figure respectively, Isabelle’s automation provides a simpler proof which does not make any use of other deductions, similar to proofs found for the deductions of the First Figure (code screenshot 3), thus Baroco and Bocardo appear as perfect deductions too, such as these of the First Figure. According to Aristotle’s proofs as indicated by Smith (2019) (see Tables 5 and 6), Baroco and Bocardo would not even be proved via direct proof, but only via an indirect proof (i.e., by impossibility), following the procedure shown in code screenshot 11. Moreover, as we will comment in the next subsection, Baroco and Bocardo, together with Barbara, are the only deductions that do not have multiple direct proofs.

### Finding all Alternative Direct Proofs

Here we note that apart from formalising the Figures by the use of some available proofs, Isabelle can be further used to find all the possible alternative direct proofs by which a certain Mood can be reduced to another, which are described in Wapniarski and Urbański (2024a).

In code screenshot 15 all the possible alternative direct proofs found by Isabelle for the Second Figure deductions are shown, which correspond exactly to those reported in Wapniarski and Urbański (2024a). All the alternative proofs were found by automation alone, just by calling Sledgehammer. In cases where not many alternative proofs were possible, a single call was enough to generate all the possibilities, given by the different automated theorem provers that are called by Sledgehammer. In cases where more proofs were possible, after hiding (commenting out in the code) the generated proofs, Sledgehammer was called again. In this way, Sledgehammer was able to find all the possible alternative direct proofs. We note that Sledgehammer had not been provided with any specific hints at all, i.e., no lemmas (no deductions, conversions, subalternations) nor proof methods were suggested to Sledgehammer by the user. The same was done for the First, the Third, and the Fourth Figure (a procedure which the reader can easily reproduce themselves). All the proofs found by Isabelle for those Figures directly correspond to those reported in Wapniarski and Urbański (2024a).

Several comments can be made. First of all, we note that we have verified the fact noted in Section 4.3 that Baroco and Bocardo (in addition to Barbara) do not have any alternative direct proofs. However, it is interesting to note that Darii and Ferio from the First Figure which also appear as perfect deductions, do have different alternative direct proofs. Moreover, from code screenshot 15 we can see that all the proofs of Moods which require some existential assumption make use of either the third conversion rule or one of the subalternations (which need existential assumptions in their own proofs), or of a Mood which itself needs an existential assumption in its own proof, as expected. This is also true for proofs found for deductions in the other Figures. In this way, Isabelle proves to be of practical use both for finding all possible alternative proofs and for finding all cases where an existential assumption is needed, with all its findings agreeing with those reported in Wapniarski and Urbański (2024a) and Wapniarski and Urbański (2024b) (note the exception of the direct and perfect proofs for Baroco and Bocardo as discussed in Sect. 4.3, which appear to be a novel finding of Isabelle).

**Fig. 15:**
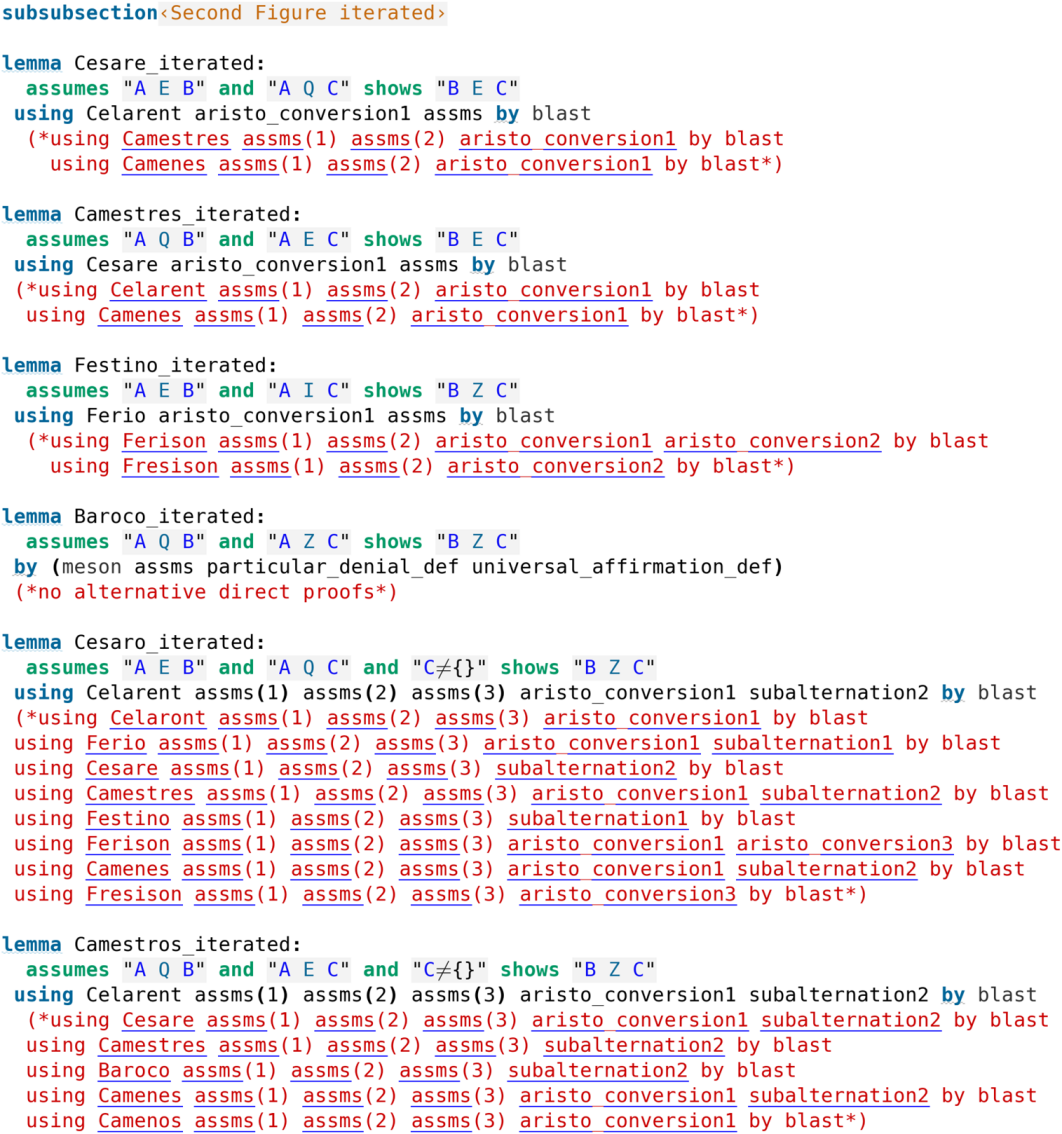
All possible alternative direct proofs for the deductions in the Second Figure

### Length and Presentation of the Formal Proofs

The main proof development (Koutsoukou-Argyraki (2019))is very short, consisting of approximately 200 lines of code including comments and examples (not including the small extension presented in Sect. 3). The de Bruijn factor (Wiedijk (2000)) is a rough measure of how much longer a formalised proof is compared to the corresponding informal one: it is defined as the ratio of the number of lines of the formal proof over the number of lines of the corresponding informal one. In mathematics, due to the high level of detail in which a mathematical proof must be “explained” to a computer, the formalised proof is usually much longer, with de Bruijn factors usually between 4 and 25 or even more. However, here we can see that the formal proofs are just one-line proofs, much shorter than Aristotle’s original proofs, as here the set-theoretic formulation makes the proofs very simple in essence, and thus the reasoning can be “packed” in the automation, which provides proofs by Isabelle’s various inherent proof methods. Thus the de Bruijn factors here can be considered to be much less than 1. For example, compare Aristotle’s verbose proof of Camestres as explained by Smith (2019):

“If A belongs to every B (:= every B is A) but to no C (:=no C is A), then neither will B belong to any C (:=no C is B). For if A belongs to no C (:= no C is A), then neither does C belong to any A (:= no A is C); but A belonged to every B (:=every B is A); therefore, C will belong to no B (:= no B is C) (for the First Figure has come about). And since the privative converts, neither will B belong to any C (:=no C is B).”^4^

with the above presented one-line Isabelle proof of Camestres (see code screenshot 5 – as we have noted before, Isabelle’s proof of Camestres presented here uses Cesare instead of Celarent, however Cesare reduces to Celarent).

It can be argued that, as true understanding is what is sought, the user may not be satisfied by Isabelle’s one-line proofs where the underlying chain of reasoning may seem cryptic, since step-by-step reasoning is packed inside Isabelle’s automatic proof methods. However, Isabelle’s formal proofs can still serve as a reassurance that a certain argument is provable (as long as the user has faith in Higher-Order Logic on which Isabelle/HOL is built) while at the same time they provide at one glance an indication about which ingredients are needed for a proof, thus guiding the user to reconstruct the proof by hand by themselves, if they wish.

## Conclusion

We have presented a formalisation of Aristotle’s Assertoric Syllogistic in the proof assistant Isabelle/HOL (Koutsoukou-Argyraki (2019)) and discussed some observations. In conclusion, we have seen that the process of formalisation with Isabelle/HOL provided us with valuable assistance in reproducing and studying the deductions in Aristotle’s Assertoric Syllogistic and relevant proofs and metatheoretical ramifications. The interactive nature of the proof assistant and its powerful automation could support and facilitate our various metatheoretical explorations, including finding proof reductions, determining wherever existential assumptions are necessary, and finding all possible alternative proofs of each deduction, thus demonstrating that Isabelle/HOL can be used as a valuable tool to study, explore, understand, and teach topics in logic and philosophy.

## Data Availability

Not applicable.

## Footnotes

1 https://isabelle.in.tum.de/.

2 https://www.isa-afp.org/.

3 For a complete list see https://www.isa-afp.org/topics/logic/philosophical-aspects/

4 The chain of arguments here is as follows: We assume AQB and AEC and we want to prove BEC. We deduce CEA from AEC and the first conversion rule. From AQB and CEA, Celarent of the First Figure gives CEB and, by applying the first conversion rule, we deduce BEC.
